# Intestinal Alkaline Phosphatase Interaction Gut Model

**DOI:** 10.1007/s11538-026-01727-y

**Published:** 2026-09-28

**Authors:** Eric Scott, Siddhartha Ghosh, Angela Reynolds, Rebecca Segal

**Affiliations:** 1 Department of Mathematics and Applied Mathematics, Virginia Commonwealth University, 1015 Floyd Ave, Richmond, VA 23284 United States; 2 Department of Internal Medicine, Virginia Commonwealth University, 1101 E. Marshall St, Richmond, VA 23284 United States

**Keywords:** IAP, Atherosclerosis, Inflammation

## Abstract

High fat Western diet (WD) is known to alter the composition of gut microbiota causing the release of lipopolysaccharide (LPS) (Cani et al. 2007). LPS is a TLR4 ligand, produced by Gram-negative bacteria, and is a potent inducer of various inflammatory cytokines. Inflammation in the gut can promote leaky gut syndrome which results in the translocation of LPS from the gut to circulation. The resulting leakage activates pro-inflammatory cytokines and alters macrophage polarization (Pelegrin and Surprenant 2009). This systemic inflammation can aggravate various diseases such as diabetes, atherosclerosis and chronic liver disease. Intestinal Alkaline Phosphatase (IAP) dephosphorylates LPS and renders it inactive, subsequently reducing leaky gut phenomenon. Informed by mouse model data, we developed an ordinary differential equation model to dynamically model the effect of diet on circulating LPS levels. The model allows for better understanding of systemic risk factors and for exploration of potential treatment using IAP-based interventions.

## Introduction

Complications resulting from systemic inflammatory diseases make them collectively the leading cause of death worldwide (Furman et al. 2019). Included in this total is the estimated yearly death toll from cardiovascular disease at nearly 18 million (Hamczyk and Andrés 2018). Although many factors are involved in the development of systemic inflammation, immune cell products are particularly significant given their role in driving inflammation processes.

This work focuses on primary players related to inflammation in the gut of mice during exposure to different diets. This work is motivated by the desire to understand the role diet plays in driving systemic inflammation linked to diseases, including atherosclerosis, and the potential to reverse this driver of the disease. We focus on a few key cell types whose activities are known to impact systemic inflammation. Macrophages are immune cells which can polarize to a pro-inflammatory (M1) or anti-inflammatory (M2) phenotype. The balance of this polarization is critical in maintaining a healthy level of systemic inflammatory response.

**Fig. 1:**
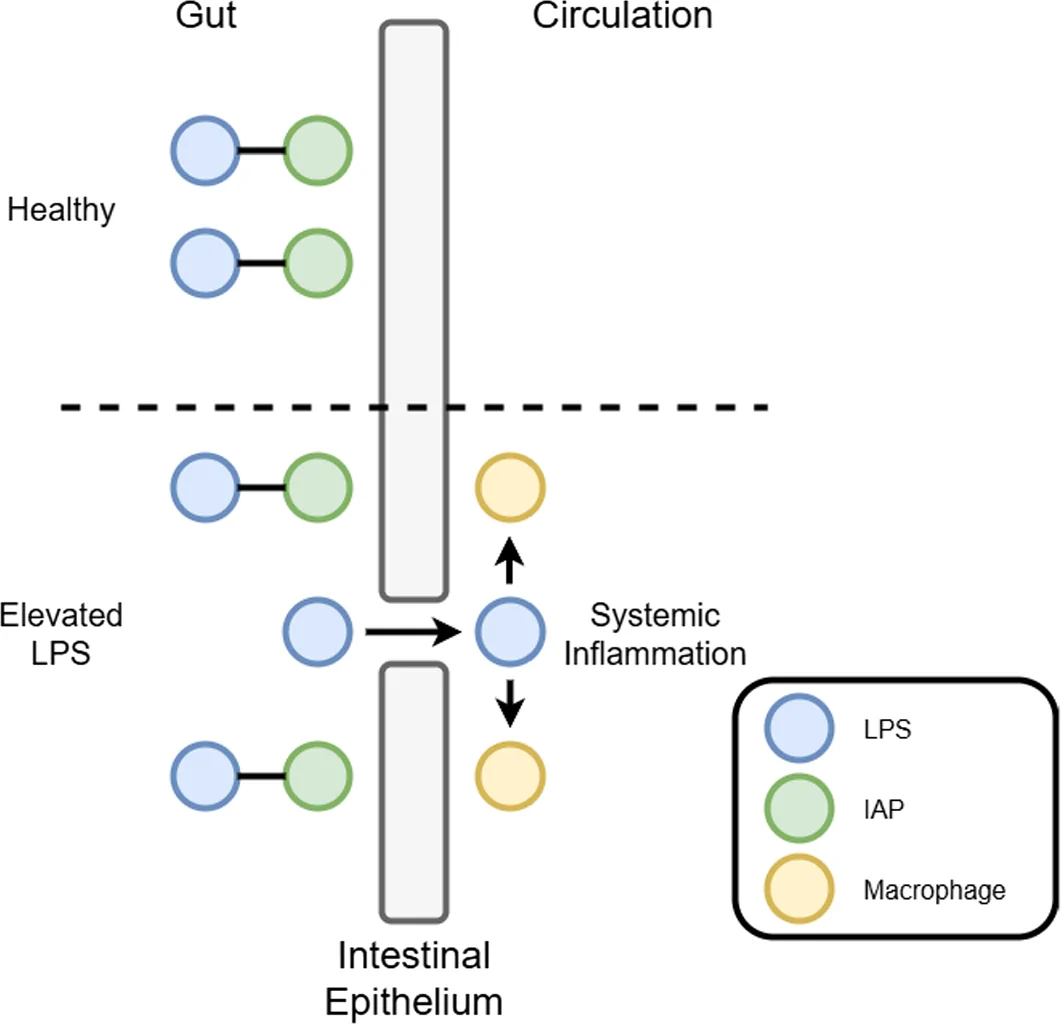
Model diagram representing biological behavior for healthy and elevated levels of Gut LPS (colour figure online)

In order to track a driving signal for inflammation, lipopolysaccharide (LPS) is measured. LPS is a bacterial component which exists naturally in the gut microbiome. It is detoxified by the enzyme intestinal alkaline phosphatase (IAP), preventing it from escaping through the gut lining and entering the bloodstream. A high-fat Western diet (WD) is believed to lower IAP activity and contribute to systemic inflammation by weakening the gut lining and destabilizing a healthy microbiome, which allows trace amounts of LPS and cholesterol into the blood, inducing or exacerbating the inflammatory response and disturbing the balance of macrophage polarization, depicted in Fig. 1. While low levels of plasma LPS are to be expected even with a normal gut, it has been demonstrated that WD causes a several-fold increase in circulating LPS in mice (Ghosh et al. 2014). It has also been demonstrated that increased levels of IAP attenuates the transmission of LPS into circulation (Ghosh et al. 2021).

Several differential equation models have depicted elements of inflammatory progression in plaque arterial formation (Bulelzai and Dubbeldam 2012; Calvez et al. 2010; Chalmers et al. 2015; Cobbold et al. 2002; Friedman and Hao 2015; Ibragimov et al. 2005; Ougrinovskaia et al. 2010; Thon et al. 2018). Torres (2019) incorporated and built on these previous models, arriving at a model which includes macrophage phenotypes (pro- and anti-inflammatory), neutrophils (normal and apoptotic), foam cells, cholesterol in plaque, and cholesterol in foam cells (free and esterified). It shows the mechanisms of macrophage polarization, accumulation of cholesterol esters, formation of foam cells, and reverse cholesterol transport. Thon et al. (2019) developed a spatial model of early inflammation incorporating the physics of blood flow and shear stress. Thon et al. (2018) also developed a submodel depicting the process of reverse cholesterol transport by which macrophages can transfer free cholesterol to High Density Lipoprotein (HDL). Chalmers et al. (2015) demonstrates the importance of the inflammatory response and macrophage polarization in inflammatory progression. The role of the immune response is explored in detail by Hansson and Libby (2006), including the complex pro- and anti-inflammatory mediators at work. In the context of the gut, Wendelsdorf (2011) has explored inflammation regulation specifically in Inflammatory Bowel Disease, focused on the mechanisms of macrophage activation as a target for intervention strategies. Arciero et al. (2010) has used ordinary differential equations to model gut inflammation as a variable in exploring probiotics as a treatment for necrotizing enterocolitis. Prana et al. (2019) has modeled the link between long term high calorie intake and inflammation resulting from increased adipose mass. In relation to the models described above, our model focuses on dietary input to the gut, and specifically the mitigating contribution of IAP, to better understand the progression and resolution of the inflammatory processes. Stamatopoulos et al. (2025) have used specialized commercial software Simcyp and GastroPlus to build a PBPK model to explore IAP as a critical factor in the performance of phosphate prodrugs. They use ODEs to model conversion rates of prodrug and include the effects of IAP on the clearance of prodrug. Relouw et al. (2024) use ODEs to model LPS-induced inflammation in liver disease. An ODE gut model by Franks et al. (2024) explored the relationship between the gut microbiome, intestinal barrier, and immune response, modeling the beneficial effects of vitamin D as an inflammatory mitigator. In our model IAP plays a similar role to Vitamin D. Gao et al. (2023) have reviewed the protective effects of IAP on the intestinal barrier. They note the phenomenon of increased LPS translocation in several disorders and call for further research into the use of IAP as a protective dietary supplement. Our gut model depicts that translocation and explores the effects of IAP supplementation as a mitigating factor.

In the sections below we describe a differential equation model for inflammation due to gut dysfunction, fit the model to experimental data, and use the model to show possible outcomes with different interventions. By modeling LPS transport into the blood as a result of diet, our model can be used as a submodel in many of these models to create a more comprehensive picture of the systemic processes involved.

## Model Development

**Fig. 2:**
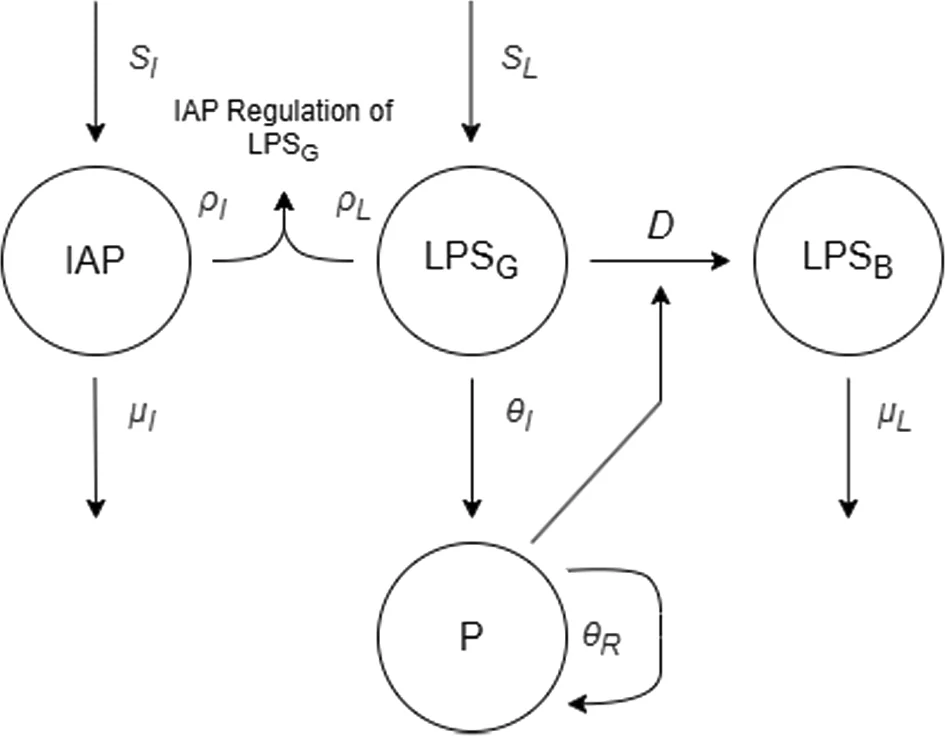
Model diagram representing interactions between variables

We model the interaction between IAP and LPS and the resulting effect on gut permeability. While this gut model focuses on the effect of WD on LPS accumulation in the blood, future work will explore this accumulation as a dynamic input to a broader systemic model exploring the effects of WD on macrophage polarization and the progression of systemic inflammation. The gut model tracks the dynamics of IAP and LPS present in both the gut and blood. Permeability is qualitatively modeled as a response to diminished IAP, and functions as a diffusion modifier for transfer of LPS from the gut to the blood. The resulting gut model consists of four ordinary differential equations with thirteen parameters. These four equations are adjusted across multiple dietary scenarios as described below. The variables we track in this model are IAP activity (*IAP*), permeability (*P*), gut LPS ($LPS_G$), and blood LPS ($LPS_B$). The model is developed by mathematically representing the significant biological effects at work in the gut and calibrating the parameters to the available data as described in the Model fitting section. A diagram showing these variables is shown in Fig. 2.

### Model Equations

IAP specific activity is defined as mmoles PNP released/h/mg protein. Experimental data estimates IAP activity as a percent of chow-fed control data. We use raw measurements taken from mouse ileum (Ghosh et al. 2018) to determine where the approximate IAP baseline level should be, and align model behavior of IAP activity with the fold-change seen in Ghosh et al. with WD (Ghosh et al. 2014). To model IAP activity (*IAP*) we developed Equation 2.1, which is comprised of a source term, interactions with the LPS in the gut ($LPS_G$) and a decay term.

2.1 $$\begin{aligned} \frac{d[IAP]}{dt}&= \overbrace{{\alpha }{S}_I}^{\text {source}}-\overbrace{{\rho _I}{[IAP]}\,{[LPS}_G]}^{\text {IAP-LPS interaction}}-\overbrace{\mu _II}^{\text {decay}} \end{aligned}$$

IAP is an enzyme produced in cells of the intestinal lining (Ghosh et al. 2021). This production is modeled as a linear source term and is the first term in Equation (2.1). To capture the overexpression of IAP in IAPTg mice this term is altered. For a WT mouse, the source term is unmodified (α=1). For an IAPTg mouse, the source term is multiplied by α>1, representing overexpression of IAP.

The second term in Equation (2.1) models the interaction between IAP and gut LPS. This term captures the decreased IAP activity observed when Western diet causes an increase in gut LPS. IAP mitigates LPS movement into the bloodstream by binding with the LPS. Therefore, IAP decreases as gut LPS increases. The decay term (last term in Equation (2.1)) represents the reduction in IAP activity from clearance of IAP from the gut. IAP is cleared by natural breakdown driven by a short half-life (Pickkers and Snellen 2009; Young et al. 1981), and also undergoes translocation from the intestine during fat absorption (Hansen et al. 2007; Inglis et al. 1967). The decay term of Equation (2.1) primarily captures natural enzymatic decay.

To account for change in intestinal permeability *P*, we developed a qualitative measure of gut health which serves as a valve regulating LPS movement from the gut into the bloodstream. We modeled the dynamics of this variable using Equation (2.2).

2.2 $$\begin{aligned} \frac{dP}{dt}&= \overbrace{\theta _I\left( \frac{[LPS_G]}{X_{ad}+[LPS_G]}\right) }^{\text {increase due to higher gut LPS}}-\overbrace{\theta _{R}\frac{P}{P+K_R}}^{\text {recovery}} \end{aligned}$$

Increases in permeability are modeled in the first term of Equation (2.2) such that small fluctuations in IAP activity do not trigger an immediate significant change, but a significant decrease in IAP activity causes permeability to rise. We use a Michaelis-Menten term to simulate this behavior with $X_{ad}$ determining the level of $LPS_G$ at which the term reaches half its maximum rate. The second term in (2.2) is a nonlinear recovery term to allow for improvement of permeability as IAP levels return to normal. Here we use a Michaelis-Menten type term with with $K_{R}$ determining the level of *P* at which the term reaches half its maximum recovery rate.

As a diet is consumed there are changes in the gut LPS as it interacts with IAP and translocates to the blood. These changes in gut LPS are modeled using Equation (2.3).

2.3 $$\begin{aligned} \frac{d{[LPS_G]}}{dt}&= \overbrace{{\beta }{S_L}}^{\text {source}}-\overbrace{{\rho _L}[IAP]\,{[LPS}_G]}^{\text {IAP-LPS interaction}}-\overbrace{P({[LPS_G]}-{[LPS_B]})}^{\text {translocation to blood}} \end{aligned}$$

These dynamics include a source term, an interaction term with IAP similar to Equation (2.1), and movement of LPS out of the gut. The presence of WD alters this equation by multiplying the source by β>1, representing increased LPS and corresponding pressure on IAP activity, while for chow diet the source term has β=1. Interaction between LPS and IAP in the gut binds LPS such that it cannot move into the blood. This is modeled with the second term in Equation (2.3), which has the same form as the second term in Equation (2.1) but with a different rate constant, $\rho _L$. Gut LPS is also reduced by translocation to the blood. We model this translocation using a diffusion term with a rate determined by *P*. Increases in *P* cause the LPS in the gut to more easily move into the blood. Given the size differences in the compartment, the concentration of LPS in the blood is less than the concentration LPS in the gut. Therefore, this term gives rise to a movement of LPS into the blood.

The final equation, Equation (2.4), models the blood LPS levels ($LPS_B$).

2.4 $$\begin{aligned} \frac{d{[LPS_B]}}{dt}&= \overbrace{DP({[LPS_G]}-{[LPS_B]})}^{\text {permeability-induced increase}}-\overbrace{{\mu _L}{[LPS_B]}}^{\text {decay}} \end{aligned}$$

The blood LPS levels rise when intestinal permeability rises, allowing transfer of LPS from the gut compartment to the blood compartment. This is modeled as in the gut LPS equation, Equation (2.3), but with an additional parameter, *D*. *D* accounts for differences in concentration change in one compartment compared to the other due to differences in compartment sizes. LPS in the blood may be removed by HDL uptake, clearance by the liver, or pro-inflammatory macrophage polarization. We account for LPS removal with a linear decay term, the last term in Equation (2.4), though if used in a systemic model accounting for these processes, this decay term could potentially be replaced by those dynamics.

We recreate four experimental scenarios: Wild Type mice with and without Western diet (α=1, β>1 with WD and β=1 with normal chow), and IAP-Transgenic mice with and without Western diet (α>1, β>1 with WD and β=1 with normal chow). Experimental data used to calibrate the model is provided in Table 2, and includes data for IAP activity, permeability, and blood LPS.

We arrive at the following model for IAP-LPS interactions, Equation (2.5-2.8).

2.5 $$\begin{aligned} \frac{d[IAP]}{dt}&= {\alpha }{S}_I-{\rho _I}{[IAP]}\,\,{[LPS}_G]-\mu _II \end{aligned}$$

2.6 $$\begin{aligned} \frac{dP}{dt}&= \theta _I\left( \frac{[LPS_G]}{X_{ad}+[LPS_G]}\right) -\theta _{R}\frac{P}{P+K_R} \end{aligned}$$

2.7 $$\begin{aligned} \frac{d{[LPS_G]}}{dt}&= {{\beta }}{S_L}-{\rho _L}[IAP]\,\,{[LPS}_G]-P({[LPS_G]-[LPS_B]}) \end{aligned}$$

2.8 $$\begin{aligned} \frac{d{[LPS_B]}}{dt}&= DP({[LPS_G]-[LPS_B]})-{\mu _L}{[LPS_B]} \end{aligned}$$

Table 1 includes a description of model parameters. Parameter values shown are from the final parameter fit, described below. Permeability in this system is unitless, so some parameters are as well.

**Table 1:** Model parameters

$S_{I}$	IAP source: WT mouse	15.92 mmoles PNP released/h/mg protein/week
$S_{L}$	LPS source on chow diet	1.585 EU/mL/week
α	IAP source multiplier: IAPTg	2.428
β	LPS source multiplier on WD	14.81
$\rho _I$	IAP-LPS interaction	0.07045 $\textrm{week}^{-1}$/EU/mL
$\mu _I$	Decay rate of IAP	2.700 $\textrm{week}^{-1}$
$\mu _L$	Decay rate of blood LPS	0.2296 $\textrm{week}^{-1}$
$\rho _L$	IAP-LPS interaction	0.04564 $\textrm{week}^{-1}$/mmoles PNP released/h/mg protein
$X_{ad}$	Triggers permeability response	2.445 mmoles PNP released/h/mg protein
$\theta _I$	Scales increase in permeability	8.880
$\theta _R$	Rate of permeability recovery	21.02
$K_R$	Limiter on rate of recovery	0.1518
*D*	LPS response to permeability	0.001875 EU/mL/week

### Model fitting

Model fitting is conducted in Matlab, with deviation from experimental data set as an objective function minimized using the fmincon function. The fmincon function calls a custom function which returns an objection function value giving the overall deviation from experimental data resulting from the parameter set provided. Each starting parameter set was adjusted based on fmincon’s interior-point algorithm before reaching an optimized set or reaching the maximum function evaluations (3000) or maximum iterations (1000). Deviation from experimental data is defined as the sum of squared percent errors from model output to target data. Within the minimize function, the system of ODEs is solved using the ode15s solver, with the ODE options set to a maximum step size of 0.1, relative tolerance of 0.01, and absolute tolerance of 0.01. Several safeguards are applied within the minimize function to protect against computationally unhealthy parameter sets. The minimize function runs the chow diet system to steady state before transitioning to Western diet. An initial optimization pass, in which all thirteen parameters are allowed to vary freely, is completed using parameter bounds set according to biological feasibility. Subsequent refinement is conducted after parameter sensitivity analysis to determine final fit.

Model development utilizes experimental data from Ghosh et. al collected from two strains of mice (Ghosh et al. 2018): IAP Transgenic (IAPTg) mice developed to express increased levels of IAP and Wild Type (WT) mice as a baseline. In one experiment (Ghosh et al. 2014), WT mice were fed a high fat, high cholesterol Western diet (TD.88137) for sixteen weeks. Plasma LPS levels and IAP activity were measured at the end of the feeding period and compared to chow diet (CD) controls. In another experiment (Ghosh et al. 2021), IAPTg mice underwent a similar high fat feeding program and it was shown that the increased IAP activity in IAPTg mice mitigates the effect of WD on blood LPS levels. Ghosh et al. (2018) also observed that fecal zonulin levels, an indicator of intestinal barrier dysfunction, decreased by approximately 50 percent in IAPTg mice given WD compared to WT mice.

Our model simplifies IAP and LPS generation to a single source term for each (with a multiplier for mouse type and diet). IAP-controlling parameters are calibrated by the ability of the model to reach the target data (Table 2). Parameters in LPS Equations (Equation 2.3 and 2.4), like β, are calibrated by the ability of the model to reach the Western diet target data. Permeability in this model is a dimensionless variable that acts as a regulator of LPS transit. We do not have data quantifying permeability directly. Therefore, the structural parameters guiding Permeability behavior are also set according to desired model behavior rather than direct reference. In particular, $X_{ad}$ and $K_r$ are set such that low-level LPS transit occurs even on chow diet, given that gut permeability is never perfect. This assumption guided model calibration by requiring Permeability parameters to always allow some degree of transit rather than disabling the blood LPS equation in the absence of Western diet. Additionally, they are calibrated to be responsive to Western diet.

We use this data and assumptions on permeability and LPS to calibrate a model to the experimental observations. The resulting model is able to capture the two variants of mouse and the presence or absence of WD by the modification of a minimal number of parameters. These four scenarios (WT mouse on CD, WT mouse on WD, IAPTg mouse on CD, IAPTg mouse on WD) are fitted simultaneously to experimental data.

Experimental data used to aid model calibration is provided in Table 2. The targets for IAP and Blood LPS are either direct experimental measurement or derived from direct measurement. “Pre-diet" refers to pre-Western diet, i.e. chow diet. “Post-diet" refers to a mouse which has been on WD for sixteen weeks. Pre-diet targets refer to steady state values, while post-diet targets are for sixteen weeks after beginning WD in a system which was at pre-diet steady state. The best fit values for these target measurements are provided in the last column of Table 2. The parameter sensitivity analysis used to determine the parameter values that have given rise to this fit is described in the next section.

**Table 2:** Target data

Target $\textrm{Name}^{5}$	Target Value	Source	Fit Value
IAP (CD, WT)	5.136 mmoles PNP released/h/mg protein	Ghosh et al. (2014)^1^ Ghosh et al. (2018)^2^	5.034 mmoles PNP released/h/mg protein
IAP (WD, WT)	1.284 mmoles PNP released/h/mg protein	Ghosh et al. (2018)^2^	1.412 mmoles PNP released/h/mg protein
IAP (WD, IAPTg)	5.877 mmoles PNP released/h/mg protein	Ghosh et al. (2018)^2^	5.228 mmoles PNP released/h/mg protein
Permeability (WD, WT)	2.61*$P_{WT}$	Ghosh et al. (2018)^3^	1.69*$P_{WT}$
Permeability (WD, IAPTg)	2.27*$P_{IAPTg}$	Ghosh et al. (2018)^3^	2.57*$P_{IAPTg}$
Blood LPS (WD, WT)	0.0908 EU/mL	Ghosh et al. (2018)^4^	0.09770 EU/mL
Blood LPS (WD, IAPTg)	0.0585 EU/mL	Ghosh et al. (2018)^4^	0.05341 EU/mL

^1^ Figure 1C and Results statement “Western diet feeding significantly reduced IAP activity (approx. ↓75%)”. Multiplied footnote 2’s value (1.284) by 4. This is only for WT mice.

^2^ Figure 2B provides WD IAP values for both mice. Post diet IAP value for WT is used to derive pre-diet IAP value for WT in conjunction with footnote 1. Pre-diet IAP value for IAPTg is not available as the same 75 percent decrease can’t be assumed.

^3^ Figure 5A. P represents pre-diet permeability. Pre-diet permeability is not a fixed target and only provides the derivation of post-diet target.

^4^ Figure 5C provides post-diet blood LPS for both mice. Ghosh et al. (2014) provides pre- and post-diet LPS for WT mouse only, but the numbers are extremely different so they are excluded, since Ghosh et al. (2018) is used for virtually all of the rest of the target data.

^5^ CD refers to chow diet, i.e. pre-Western diet. WD refers to Western diet. WT refers to Wild Type mouse. IAPTg refers to IAP Transgenic mouse

### Parameter sensitivity

The four possible system configurations require simultaneous fitting of thirteen parameters across sixteen ODEs (four variants of a four-equation system). However, the number of varying parameters may be reduced as described below.

Evaluating the parameter sensitivity identifies opportunities for improving model fit. After the initial optimization pass is complete, we fix a subset of parameters based on sensitivity and collinearity.

Parameter sensitivity analysis is performed using Matlab’s Simbiology tool. After the initial fitting algorithm in which all thirteen parameters vary freely, local sensitivity *S* is determined for each parameter using Root Mean Squared calculation (2.9) on the Simbiology output, where $s_{i}$ is the raw output of Simbiology’s calculation of that parameter’s partial derivatives (Equation 2.9).

2.9 $$\begin{aligned} S=\sqrt{\frac{\sum _{i=1}^{n} s_{i}^2}{n}} \end{aligned}$$

**Fig. 3:**
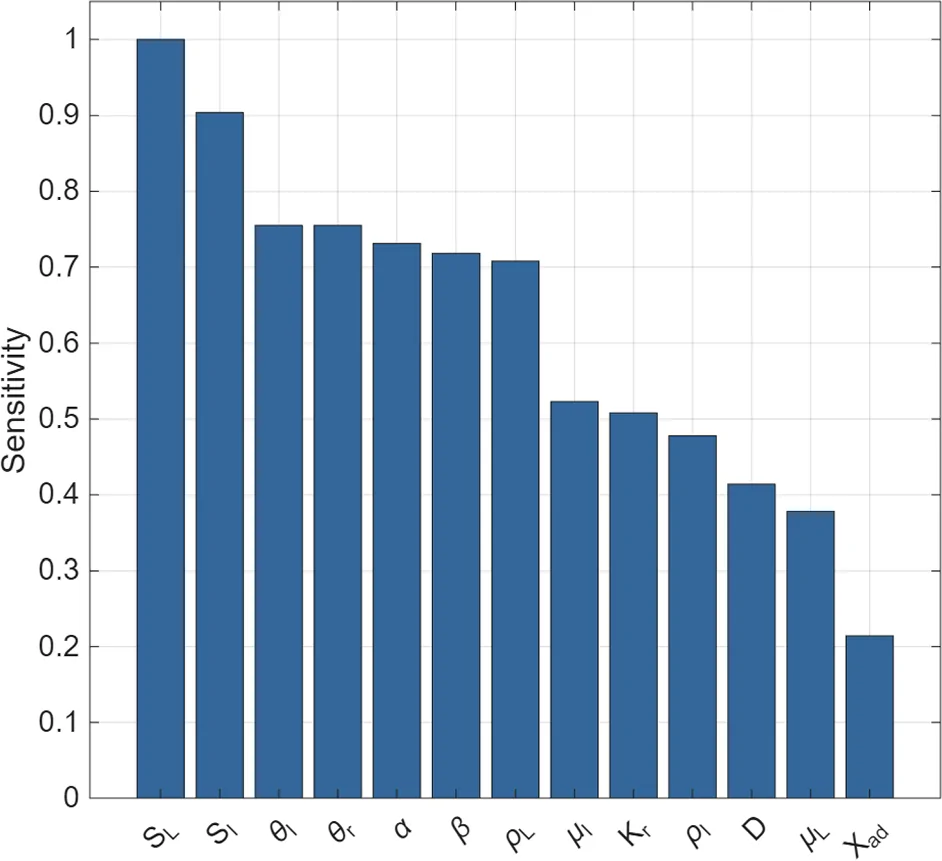
Normalized parameter sensitivity. Represents overall sensitivity across all four combinations mouse and diet

Since this system transitions from chow diet to Western diet, the end state of chow diet (i.e., the beginning state of Western diet) may influence the end state of Western diet, and we include Initial Conditions as sensitivity outputs from Simbiology. The Initial Conditions showed near-zero sensitivity, confirming no significant influence. The thirteen parameter sensitivities were then normalized. The results of this initial sensitivity analysis are shown in (Fig. 3).

The results of the sensitivity analysis show that none of the thirteen parameters fall below a clear insensitivity threshold (normalized sensitivity of 0.1 was used as the target threshold), with the least sensitive parameter still greater than 0.2 normalized sensitivity. However, the sensitivity matrix calculated for the same parameter set used in Simbiology does offer useful information. To begin, the condition number is extremely high (on the order of 1e13). Two parameters, $\theta _I$ and $\theta _R$, have essentially identical absolute values for sensitivity. These parameters scale the increase and decrease of Permeability and appear nowhere else in the system. Their similar sensitivities contribute significantly to the high condition number, and we fixed $\theta _R$; the resulting matrix had an improved condition number.

We also examine pairwise correlation, and find very high correlation between $\mu _L$ and *D*, and between $\theta _I$ and $K_R$. Removing $\mu _L$ and $\theta _I$ lowers the condition number to 4.7e8. This is low enough to remove numerical instability, and a collinearity script is run on the remaining parameters, removing $\mu _I$ and $\rho _L$ and reducing the condition number to 4.3e2.

The resulting matrix represents eight remaining parameters and is full rank, which allows for a refinement pass of the fitting routine using fewer parameters without sacrificing behavioral insights. The five parameters which were identified in the sensitivity and collinearity assessment are fixed in the fitting routine, with the remaining eight being allowed to vary, and the initial thirteen-parameter fit being used to calibrate the starting point of the refining fit. Final parameter values are provided in Table 1.

## Results

### Model Transients

**Fig. 4:**
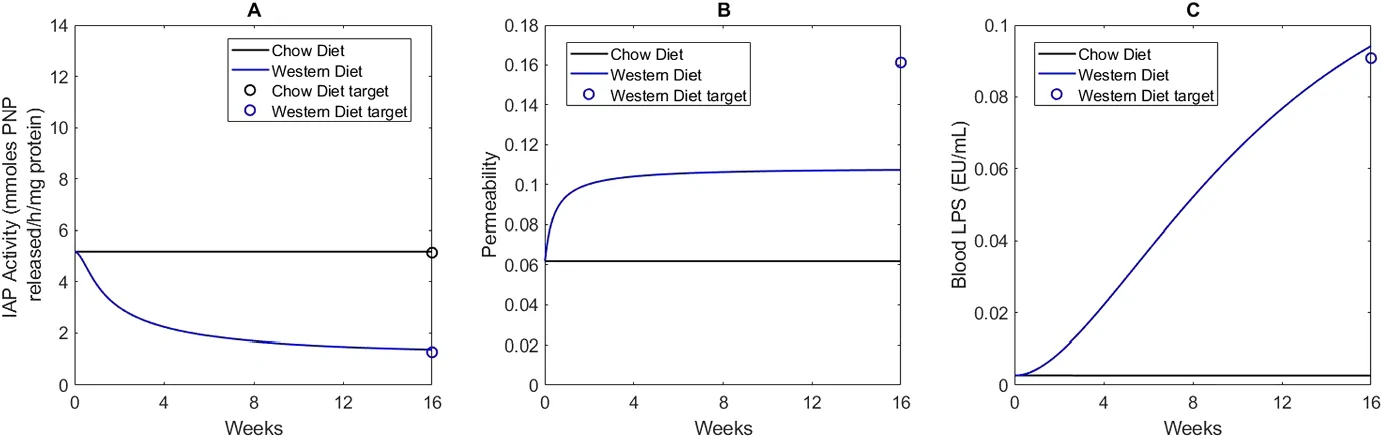
Western diet effect on IAP (A), permeability (B), and blood LPS (C) for Wild Type mouse. Using the parameter values give in Table 1 with α=1 and β equal to the value in the table (colour figure online)

**Fig. 5:**
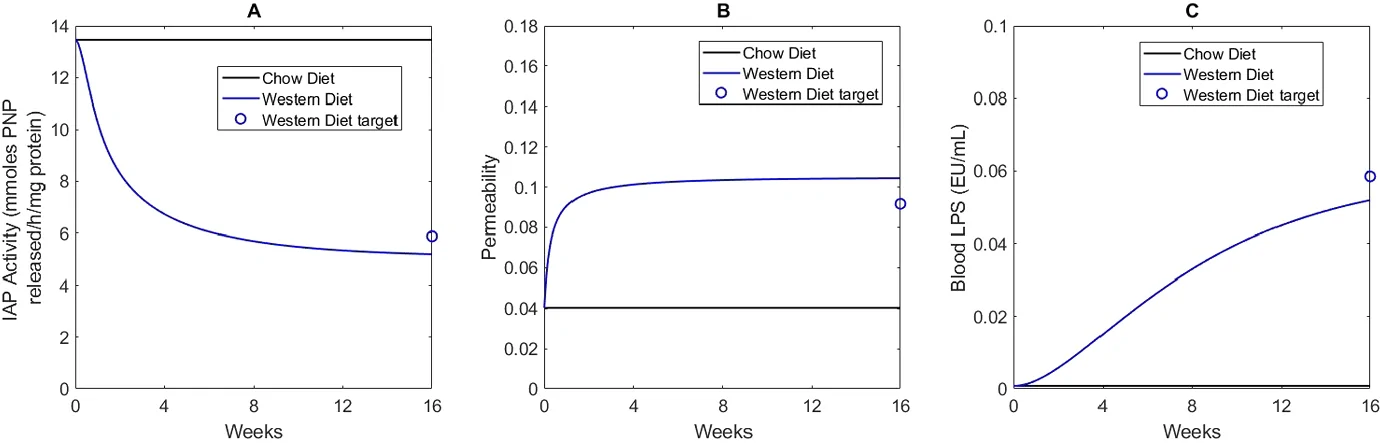
Western diet effect on IAP (A), permeability (B), and blood LPS (C) for IAPTg mouse. Using the parameter values give in Table 1 with α and β equal to the value in the table (colour figure online)

IAP decreases in response to higher Gut LPS (as would be observed on a Western diet), triggering an increase in permeability, which allows an increase in Blood LPS. Model behavior adheres well to target data derived from experiments as shown in Fig. 4 and Fig. 5.

As shown in Fig. 4, introduction of Western diet to a Wild Type mouse lowers IAP activity by approximately 75 percent, aligning with experimental data. Permeability in the model does not increase by as high an amount as the experimental target data, with this being the furthest deviation from the target. However, downstream effect on blood LPS adheres closely to experimental data - the model suggests that Western diet results in an over 30-fold increase in blood LPS, showing a substantial impact of Western diet on systemic inflammation.

An IAP transgenic mouse begins with higher baseline levels of IAP. While Western diet still causes a significant drop, the higher levels mitigate the effect; Western diet IAP levels in an IAPTg mouse are similar to chow diet IAP levels in a WT mouse. Western diet has a similar effect on gut permeability in an IAPTg mouse as in a WT mouse, even though it begins at a lower baseline. The arrival at a similar gut permeability is mitigated by the higher sustained levels of IAP, and blood LPS after sixteen weeks on Western diet is approximately half as high as in a WT mouse. The data and model suggest that increased IAP has a minor mitigating effect on the degradation of the gut lining itself, and a much more substantial mitigating effect on downstream inflammatory effects by neutralizing additional LPS before it transfers into the blood.

### Intervention Scenarios

(Ghosh et al. 2014) have shown that daily supplementation with curcumin significantly increases IAP activity and mitigates blood LPS increase due to diet. We simulate an IAP supplement by introducing additional IAP stimulus to the recovery scenarios, modeled as a once-daily additional input to IAP activity. We construct an additional stimulus to the IAP variable such that each dose decays completely before the following dose occurs given that there is no absorption of IAP.

To model this stimulus we introduced an additional variable (ψ) to our system and accounted for dosing and decay of the stimulus. This gives rise to our Equation 3.1.

3.1 $$\begin{aligned} \frac{d\psi }{dt}&= \overbrace{k\phi (t)}^{\text {drug intake}}-\overbrace{m\psi }^{\text {drug decay}} \end{aligned}$$

**Fig. 6:**
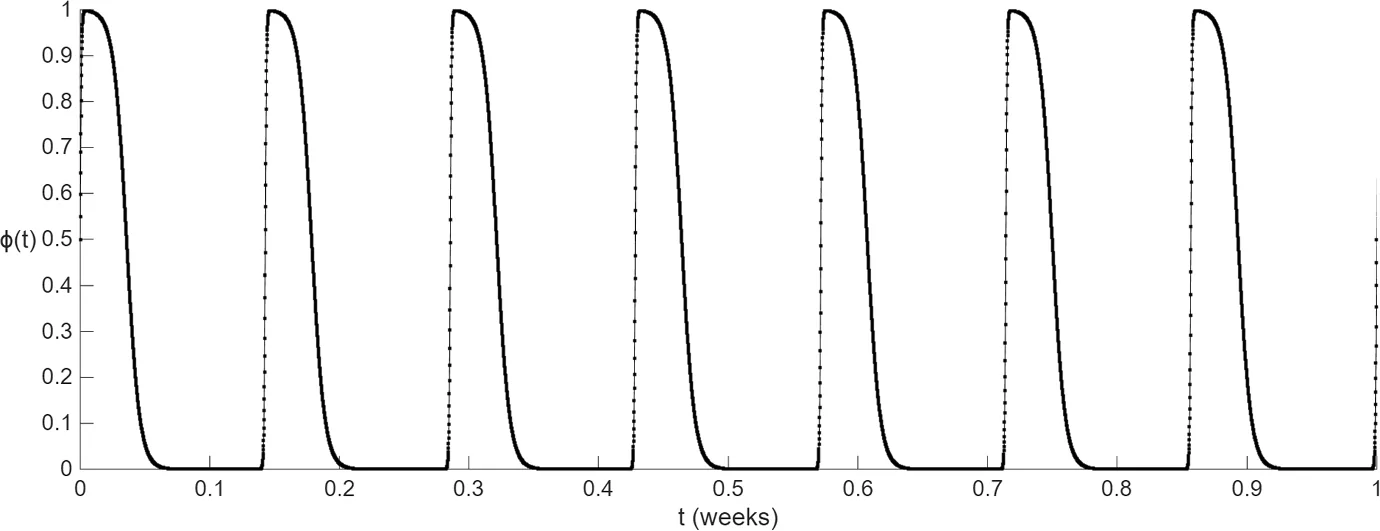
Pulsatile function ϕ(t) for generating additional IAP activity. This example shows once-daily stimulus for one week

**Fig. 7:**
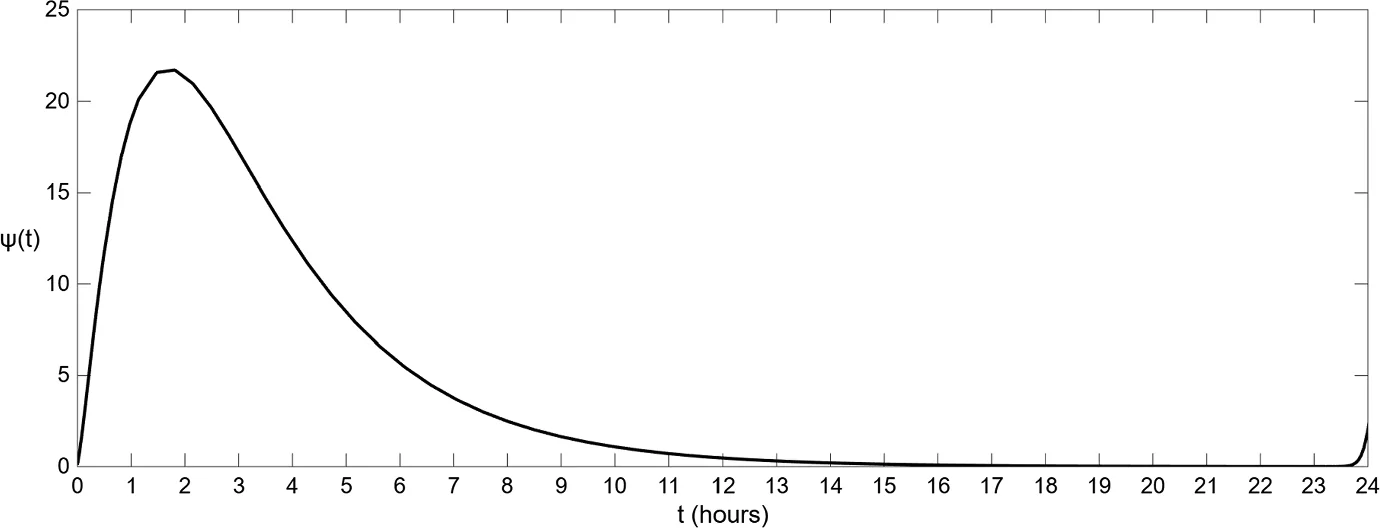
IAP stimulus function ψ(t) representing additional IAP activity. This example shows one 24-hour period corresponding to a single dose of a drug

In Equation 3.1, ϕ(t) represents daily dosing of a drug providing IAP, which causes ψ to increase, and elevated levels of ψ force IAP activity to increase. The function ϕ(t) is comprised of two logistic sigmoid functions which are multiplied together, where the exponential term in each sigmoid function contains a modulo operation in order to create a periodic pulsatile input bounded by (0, 1). The resulting function is graphed in Fig. 6. This input wave is then scaled by *k* and paired with a decay term to create the equation for the stimulus dynamics. Fig. 7 shows that stimulus over 24 hours with k=10000 and m=70.

This stimulus is then incorporated into the full model by adding ψ to the IAP equation, which gives rise to Equation 3.2.

3.2 $$\begin{aligned} \frac{d[IAP]}{dt}&= \overbrace{{\alpha }{S}_I}^{\text {source}}+\overbrace{\psi }^{\text {stimulus}}-\overbrace{{\rho _I}{[IAP]}\,{[LPS_G]}}^{\text {IAP-LPS interaction}}-\overbrace{\mu _II}^{\text {decay}} \end{aligned}$$

**Fig. 8:**
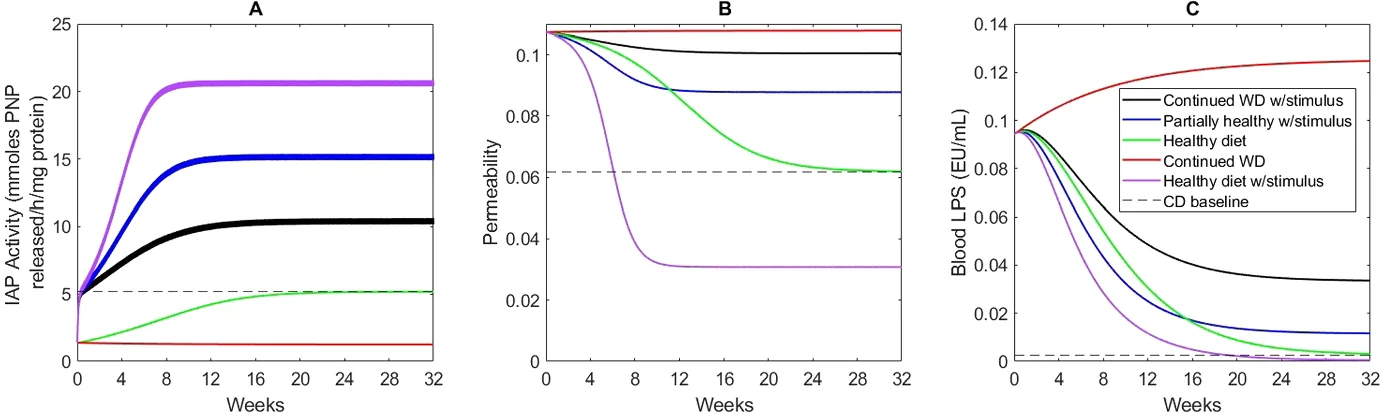
Simulated recovery of (A) IAP Activity, (B) Gut Permeability, and (C) Blood LPS after sixteen weeks on Western diet (colour figure online)

Since an individual may not exercise uninterrupted adherence to a regimen of medication and healthy diet, we simulate scenarios that account for partial adherence to healthy diet. Western diet is modeled in Equation 2.3 as β=14.81 and full adherence to healthy diet is defined as β=1; partial adherence is therefore defined by setting β to the midpoint between these two values, β=7.907. Predicted recovery scenarios with and without IAP-based intervention with both full and partial adherence are shown in Fig. 8.

The modeled recovery predicts that even full adherence to improved diet requires substantially more time to return to pre-WD levels of blood LPS than was spent on WD. Blood LPS levels approach the pre-WD baseline after approximately 32 weeks, (double the time that was spent on WD) but remain slightly elevated, even when given extended time. As Table 3 shows, healthy diet with no additional IAP stimulus results in a return to 118 percent of baseline after 32 weeks. When increasing the modeled recovery time span to 64 weeks, healthy diet with no additional stimulus finally shows a return to 100 percent of baseline, with the curve having essentially leveled off long before, as the green curve in Fig. 8 suggests. The model predicts that after 16 weeks on WD, returning to baseline within a similar 16-week time frame requires full adherence to improved diet in addition to supplemental IAP stimulus. Of particular note is the comparison between full adherence to improved diet and partial adherence to improved diet aided by IAP stimulus (the green and blue curves respectively). The model suggests that for a time, the addition of IAP stimulus outperforms fully improved diet, but after approximately 16 weeks the blue curve flattens while the green curve continues to show improvement. This suggests that IAP stimulus is an effective short term supplement while gut LPS levels are still elevated, while a fully improved diet achieves better results in the long term by addressing the root cause of the elevated LPS. However, the time at which this switch occurs is similar to the time spent on WD to begin with, suggesting that IAP stimulus is an important tool within a realistic treatment time frame.

**Table 3:** Summary of diet and IAP stimulus effectiveness after 16 weeks on WD

Regimen	Drug	Time to Recovery	Proportion of Baseline at 32 Weeks
Continued WD	Yes	-	12.53
Partially healthy	Yes	-	4.35
Healthy	No	∼38 $\textrm{weeks}^{1}$	1.18
Healthy	Yes	19 weeks	.20

While blood LPS recovery largely levels off at approximately 32 weeks onward, blood LPS returns to within 5 percent of CD baseline at approximately 38 weeks

## Conclusion

This model successfully builds on experimental data to predict the effect of WD on systemic inflammatory factors. Introducing sixteen weeks of WD to a WT mouse causes a significant increase in circulating LPS, in line with experimental data. Overexpression of IAP results in substantially mitigated levels of circulating LPS, even without significantly altering gut permeability markers.

The model shows that after 16 weeks on WD, full cessation of WD provides a return to blood LPS levels slightly elevated above baseline after roughly twice as much time, remaining slightly elevated in the absence of additional IAP stimulus. In the context of the lifespan of mice, 16 weeks represent a significant period of time even under ideal dietary conditions, and partial dietary improvement may be a realistic expectation. The model suggests that supplemental IAP stimulus provides substantial benefit in reducing circulating LPS and may even be necessary to recover LPS levels to below the pre-WD baseline in a realistic time frame. The model shows that supplemental IAP even outperforms full dietary improvement in the shorter term and serves as an immediate mediator of elevated gut LPS, while fully improved diet is necessary in the long term.

This gut model may be used with a circulatory model to dynamically depict the effect of WD on subsequent systemic inflammatory indicators, as well as the effect of IAP-based interventions on systemic inflammation. This work addresses an important input to systemic inflammatory diseases such as atherosclerosis by modeling inflammation-inducing diet as a causal vector for pro-inflammatory immune response. Increases in blood LPS caused by a Western diet stimulate plaque formation and delay recovery, even after returning to healthy diet. The model suggests that a combination of healthy diet and additional enzyme intervention is necessary in order to return blood LPS levels to a normal level once a Western diet has been active for an extended time. The effect of poor diet is long lasting and would benefit from IAP-based intervention strategies in order to mitigate the effects of diseases such as atherosclerosis.

Research shows that while IAP expression is fairly uniform along the ileum for IAPTg mice, there is a substantial dropoff in activity along the ileum in WT mice (Ghosh et al. 2018). Exploring those spatial differences could help further inform IAP intervention by examining the nature of IAP and LPS absorption along the length of the ileum and the effect of the survivability of an IAP-based drug on resulting blood LPS levels.

The ability to maintain interventions that are diet and/or medication based is challenging in treatment. Using this modeling approach to facilitate decisions about which to prioritize is not only applicable for IAP, but other drugs emerging that affect gut absorption and diet and are used to treat diabetes, heart disease and other metabolic disorders.

## Data Availability

Data used for this research is published in the works cited in Table 2 and is available in the cited works.

## Code Availability

Code for this research has been deposited on GitHub at the following link: https://github.com/scotte4vcu/IAP-Matlab
